# Extracts and Gleanings

**Published:** 1871-09

**Authors:** 


### QUININE.

BY CHARLES S. SHELDON, M. D., WINONA.

The subject I have chosen is one familiar to all. We have used this remedy daily for years, and have given more or less of study and thought to its proper administration and action in disease.

In treating this subject I do not expect to give you any discoveries, or even theories, of my own, but shall endeavor simply to review the ground gone over by others.

I shall consider briefly its action and therapeutic use.

We can not devote too much attention to the subject of therapeutics. Diagnosis and pathology have of late made great advances, and perhaps before long we shall know what we *have* to cure; but we have to admit that in the understanding of the action and agency of medicines in the cure of disease, we are not so very much superior to our ancestors. We should rise to a higher degree of enlightenment, when we may discard the empirical, and adopt the rational system of therapeutics. This can only be, till on the one hand, by an accurate knowledge of the symptoms of disease, we can meet each by its appropriate remedy, and, on the other hand, by a more definite acquaintance with the general action of a medicine, we may use it with greater skill and apply it even in cases where it has not yet proved beneficial. Especially should we study a remedy so generally used and so highly esteemed as quinine. Not a day passes but that we meet cases or conditions where its use is indicated. Questions relating to its absolute or relative efficiency in these cases, its mode of action in curing disease, its dose and manner of administration, daily suggest themselves to us, and claim our careful attention. Here in Minnesota, these considerations are especially important. We have among us those who have formerly lived under malarious influences, and there is, undoubtedly, more or less of malaria in the State. It therefore requires a nicer discrimination to ascertain those cases when quinine is indicated, and in what way to give it, than in Illinois, used heroically for ague, or in New England, used merely for its tonic effects.

As to the mode of action of quinine, there has always been, and there is now, a great diversity of opinion. Its tonic effects in some cases, its stimulant effects in others, and its sedative effects in still others, have led authors to take very different and opposite grounds. The views here presented are condensed from Headland, in his work on the action of medicines, often in his own words. I am aware that they will be met with disfavor by many, especially by those who have adopted the theory which regards the nerves as the prime factor in the production of most diseases; but they are, at least, worthy of consideration. He considers quinine as an hæmatic, and as being restorative rather than catalytic, i. e., a medicine which acts primary in the blood, to restore some material wanting, rather than to destroy a poison existing. He asks, and attempts to answer, the following questions:

1. "Does quinine act primarily in the blood, or on the nerves, and is its action of a permanent character?"

2. "Is it naturally present in the blood, or is there in the blood a substance which resembles it?"

3. "Does it remain in the blood, or is it wholly excreted?"

4. "If acting in the blood, does it effect a cure by supplying to it a material wanting, or by counteracting in it a morbid process?"

There is no doubt but that it enters the blood, since it can be detected in that fluid after its administration. Once there, does it produce its tonic and anti-periodic effects by improving the blood, and to the whole system, or does it at once, and in the first place, act on the nerves? Is it an hæmatic or a neurotic? He believes it is *primarily* a neurotic, for the following reasons: Other nerve medicines are distinguished by an action which rapidly follows their administration. This action is not permanent, but rapidly passes away. It takes place in health as well as in the use of alcohol and digitalis. Most neurotics are capable of producing action by mere external contact with the nerves. They are chiefly used when the nervous system is unusually excited or depressed, and are of no permanent benefit in diseases depending upon blood disorder.

The action of hæmatics, on the contrary, is of an opposite kind in all these particulars. When we consider the primary action of quinine, too, we must conclude that it more resembles hæmatics, and that its influence on the nervous and muscular systems is secondary. Judging from analogy in the use of neurotics and astringents, we could not expect that any permanent improvement could be effected in either nerves or muscles, without its first acting in the blood. To make the case clearer, it can be shown that the diseases in which quinine is most used, are blood diseases. *Debility* seems primarily to be due to a want in the blood, which impairs the nutrition of the nerves and other organs, and thus interferes with the performance of their functions. It follows fevers and accompanies chronic diseases, in both of which cases the blood has been exhausted by continual waste and excretion. I know that some will take exception to this doctrine. Intermittent fever, too, is now considered by most a blood disease. We might infer this from the analogy of other fevers; but we know, in addition, that it is caused by a peculiar poison, which produces in the blood a process like fermentation, causing periodical paroxysms. In the progress of the disease, too, the blood rather than the nerves, seems to be concerned. We have first a chill, then a hot stage, and then sweating. It would seem, then, as if the poison were eliminated in the sweating; but after working in the blood for a definite period, the same symptoms recur. The results of the diseases, also, are evidence of its nature. Long continued ague causes general anæmia, and an enlargement of the spleen, which could only be produced by a faulty condition of the circulation. In typhoid and typhus fevers, in scrofulous and tuberculous diseases, and in fact in all affections where deterioration of the blood is a known cause of disease, quinine, in tonic does, has the most beneficial effect. The periodical attacks of neuralgia, benefitted by the use of quinine, are generally malarial in their origin, and so due primarily to a poisoned condition of the blood. Another fact pointing in the same direction, is, that ague is not always, connected with derangement of the liver. I think these positions are, in the main, well taken by our author, and the probabilities very strong that quinine is *primarily* an hæmatic.

We now come to the next question: "Is it naturally in the blood, or is there in healthy blood any substance which resembles it? that is, is it a restorative hæmatic?" for to act as a restorative, it must take the place of something which should be present in healthy blood. Although the bitter principle of the bile seems to possess a similar character to quinine, it is only till recently that we have found proof of the existence in the blood, of a substance similar to quinine. Dr. Dupre and Dr. Bence Jones having discovered quinine in the blood of a Guinea-pig to which it *had* been administered, found precisely the same reactions in a pig to which it had *not* been given. These experiments were carefully conducted, and were conclusive as to the presence in the blood and tissues of a substance chemically identical with quinine. This they have named animal quinoidine. If this be not quinine itself, we may at least conclude that the presence of quinine in the blood, would not be unnatural to it.

*Thirdly.*—"Is quinine wholly excreted from the blood?" If not, we may conclude that it remains in it, and so can act as a restorative. From many experiments it has been proved conclusively that it does remain in the blood, i. e., when given in a small dose, it is not excreted at all, but when given in an excessive dose it makes its appearance in the urine, like other restorative medicines.

*Finally.*—"Does it improve the condition of the blood when deficient in any of its natural materials, i. e., does it act as a restorative in supplying something wanting, or as a catalytic in counteracting something present?" The fact that it is not unnatural to the blood, and is not always excreted from it, is in favor, *a priori*, of its being a restorative. Besides, a catalytic has some peculiar action on the blood in health, but a restorative, in moderate doses, none. On all these grounds we must consider quinine with arsenic, a catalytic, and we find they differ in all these particulars, and also in their use; quinine being used in debility and when there is some want in the system, arsenic in lepra and kindred skin diseases, caused by some morbid agency. To be sure both are used in ague, but perhaps this disease is curable either by supplying something, or by neutralizing something else.

If these points have been well made, we must conclude that quinine is a restorative hæmatic, and a presumtion is established that in those diseases where it is curative, there is some deficiency in the blood, which can be supplied by it. It may be said that this is simply a theory, but we accept many medical ideas as facts, which have really fewer grounds for our belief. These views are presented on the supposition that they might be new to some, and with the hope that their presentation might excite a more earnest spirit of inquiry and discussion regarding such an important topic.

Therapeutically considered, quinine is certainly one of our most valuable remedies, not only from its positive, but also from its negative virtues; for while other medicines of equal power and value, are often harmful and destructive in their character, I think we may justly class quinine among the most harmless and safe of our remedies. It is a *remedy*, and not a *poison;* and while opium and mercury have killed many, and destroyed the constitutions of more, I can find no case where quinine has produced lasting harmful results. Not but that it may be injudiciously used, especially in acute diseases, but that, on the whole, there is less danger in its use than in any other remedy of equal power. It is very unfortunate that there exists such a strong prejudice against it in the popular mind, many persons being unwilling to take it, and its beneficial effects being lessened in the case of others by their suspicions of its injurious nature. It is difficult to account for such a popular delusion; but we know that many confound cause and effect in cases of long continued ague, accompanied with the use of quinine, and that a large proportion of the community, including well educated professional men, are so grossly ignorant of its character as to suppose it a preparation of mercury or opium. We can only wait and hope that this and kindred delusions will be dissipated by time, and a greater enlightenment of those who have a moderate stock of common sense on topics disconnected with medicine. Although there is no disease in which quinine acts so favorably and surely as in malarial fevers, there is no doubt of its value in a vast number of affections. In intermittent fever the profession now favors large doses in the interval of the paroxysms, the rule being to give enough to produce a moderate degree of quinism. To stop a single paroxysm, Prof. Alonzo Clark gives gr. j. of opium and gr. x. of capsicum, with quinine. In intermittent neuralgia all authors agree that large doses, gr. xx. to gr. xxx. daily, for a week or more, will afford the best results in most cases.

The use of quinine in typhoid fever has given rise to much discussion, some claiming for it the virtues of a specific, and others stigmatising it as highly injurious. Stille concludes that it is a remedy of secondary value here, even if it possesses any virtues at all. Wood has never found in it any other favorable influence than a moderate supporting effect in the low states of these disease. Prof. Clark does not mention its use in this affection. Aiken simply says it is recommended by Dr. Mutchison in half grain doses. Flint advises its use, as a tonic, in small doses. Headland thinks it admissible in all fevers, care being taken to give it when the pulse is soft and the skin and tongue moist. Liebermeister, basing his belief on a long and carefully conducted series of experiments, thinks it has actual curative properties, in reducing the fever, and ameliorating the other symptoms. I think we may safely conclude, from our daily experience, that if it cannot claim a directly curative influence, it certainly has a tonic and supporting power, and that it will be safe and useful to give, in small doses, in nearly every stage of the disease.

In several diseases of the digestive organs it is of much service. In atonic dyspepsia, we often get excellent results from its use, either alone or with other remedies. In the night-sweats of phthisis, with sulphuric acid, it is especially serviceable, and all agree that a profuse sweating during sleep, in any disease, is an indication for its use. In convalescence from most fevers, and in all cases of general debility, it is indicated.—*Med. & Sur. Jour.*

### THE SICKNESS OF PREGNANCY: ITS CAUSE AND TREATMENT.

Dr. Hewett read a paper on this subject before the London Obstetrical Society. He remarked: The sickness observed in pregnancy has generally been accepted as an inevitable circumstance; the causes of its occasional inveterancy and even danger have never been satisfactorily made out. The treatment of these latter cases has not been conducted on any one principle; yet it must be evident that an analogous cause must be in operation in the slight cases and in the more severe forms. The present state of professional opinion may be represented in the statement that it is due to the distending effect of the increasing contents of the uterus, exciting thereby in a reflex manner the act of vomiting. The author, accepting this view, proceeds to propound the theory that the existence of flexions of the uterus in various degrees of intensity is the prime factor in giving rise to the vomiting of pregnancy in by far the majority of instances, inasmuch as it offers an additional hindrance to the proper expansion of the uterus, and gives rise mechanically to such pressure on the sensitive uterine tissue at the seat of flexion as results, in most cases, in this particular reflex irritation. This theory, as to the cause of the vomiting of pregnancy, will account for the mild and severe forms of the symptoms. The author was led to this conclusion by observation of the close connexion between obstinate nausea and vomiting, and flexion associated with distension of the uterus in the non-gravid state, as in cases of dysmenorrhœa produced by flexion. Latterly he has found himself applying this explanation to the gravid cases, and, having tested the matter by observation for some little time past, the clinical facts which he has accumulated appear very completely to bear out the general truth of the theory now enumerated. An anteflexed gravid uterus is most commonly the condition found to be present, the anteflexion existing before the pregnancy supervenes; retroflexion of the gravid uterus much less commonly, because the retroflexed uterus is less liable to become impregnated than the anteflexed organ. The very obstinate cases of sickness are observed generally at the second to the fourth month, when the uterus is sometimes found tightly fixed in the pelvis, and unable to escape from it. How far the explanation will apply to cases where the uterus is more advanced in pregnancy the author does not say, not having had cases to test the matter by. The slight cases, where the sickness is limited to to time of rising from bed, are explained by the action of gravity in the erect posture suddenly bending the uterus on itself to a slight extent. Undoubtedly whatever tends to hinder the expansion of the uterus may equally induce sickness: thus some cases may not be accounted for by the theory now put forward. The results of treatment based on the foregoing conclusions, and consisting in mearuses to restore the organ to its proper shape, have been found very successful in the author's experience—sometimes maintenance of the horizontal position alone sufficing; in other cases, mechanical supports, elevating the fundus anteriorly or posteriorly, according to circumstances, being used for the purpose. The author is quite satisfied of the value of the theory as a basis for practice. He believes that the pressure on the nerves of the uterus at the seat of the bend is the exciting agent; this pressure usually leads to congestion of the uterus above and below, and possibly to other secondary results.—*Med. & Sur. Reporter.*

---

### TREATMENT OF SORE NIPPLE.

Mr. C. Heath says, in the *Lancet:* "In the treatment of the ordinary chapped nipple, all sorts of nostrums have been advocated, but I have found nothing so satisfactory as a five grain solution of nitrate of silver, carefully pencilled on with a camel's hair brush, after each act of suckling. The employment of spirit of nitrous ether as a vehicle, instead of water, has the great advantage of giving a lotion which dries rapidly, and which is not repelled by the greasy condition of the nipple. The use of a shield if the child has an aphthous mouth, or is prone to bite, gives relief, but will not effect a cure without local medication.

Anything like violent manipulation of an engorged breast is much to be deprecated; but steady and even pressure will often given relief. It is certainly advisable to diminish the secretion of milk in the breast; and for this purpose nothing is so certain or satifactory as the external application of extract of belladonna mixed with equal parts of glycerine. The effect of belladonna, freely applied in this manner, is not merely to relieve pain, but, by causing contraction of the capillaries, to diminish the quantity of blood in the organ, and the amount of secretion. In addition to the local remedy, means should be taken to act upon the bowels; and since cases of sore nipples frequently occur in females exhausted by over-suckling, the combination of tonics with hydragogue cathartics is very useful. The formula I generally employ is, sulphate of quinine, one grain; sulphate of magnesia, four drachms; dilute sulphuric acid, five minims; pimento water, one ounce; every six hours.

If engorgement goes on unrelieved for over twenty-four hours, inflammation is pretty certain to supervene, the constitutional symptoms of which are generally well marked. If seen before the occurrence of a rigor, by which the formation of matter is generally announced, the treatment for this condition will be the same as that for simple engorgement, since one runs almost imperceptibly into the other. In addition to the use of belladonna locally, I have found the greatest benefit from the application of warmth and moisture by means of a linseed poultice placed over the belladonna; and it is most important that the breast should be thoroughly supported by a sling, or, what I prefer, a broad strap of plaster affixed to the shoulders, and carried below the organ. In debilitated patients, in whom inflammation of the breast is most generally seen, any local or general depletion is uncalled for; but the administration of the tincture of aconite, in frequent and small doses (two minims every two hours), will do much to relieve the condition of the patient, and to promote a healthy moisture of the skin.

A New Mode of Administering Copaiba.—The Lancet for April 29th ult., contains some remarks, by Mr. Berkley Hill, on the treatment of gonorrhœa, in which he recommends a form in which this remedy can be taken very easily. It resembles a jelly, is not respulsive to the taste, and if a piece the size of a hazelnut be rolled in wafer paper, it can be swallowed without being tasted at all. The after-effects, of derangement of the digestive organs, he thinks to be rather less from this than from most other forms of administering the remedy.

Take of thick copaiva, eight ounces; powdered sugar, four ounces; honey (not crystallized), four ounces; distilled water, five drachms; oil of peppermint, one drachm; roseine (one of the aniline pigments), one-tenth of a grain dissolved in twenty minims of water. Put the honey, sugar, copaiva, and water into an evaporating dish, keeping it well stirred. Heat the mixture gently till it boils, and continue the agitation and ebullition about five minutes.

In the first part of the operation, two distinct strata are formed; the upper the copavia, the lower the honey, etc. As the water is evaporated, numerous bubbles of steam are given off, just as the whole becomes a homogeneous jelly. When it has partly cooled, stir in the roseine and the oil of peppermint. When well made, it should resemble raspberry jelly. Should this very minute quantity of roseine be objected to, an ammoniacal solution of carmine gives a very good color.

### PART II.

### Abridged Extracts and Gleanings from our Exchanges

*MULTUM IN PARVO.*

CARBUNCLES are most safely, humanely and more regularly treated, as follows: Introduce the canula of a hypodermic syringe into the centre of the tumor, draw out the piston, and with it will come pus, if any. The syringe is to be removed from the canula and emptied, the canula left in, and the syringe replaced to the canula again, and the piston withdrawn, as before, as long as pus follows. When all the pus is out, withdraw the canula and apply on the tumor, externally, with a brush, the following:

| | | |
|---|---|---|
| ℞. | Collodion, . . . . . . | ℨ i. |
| | Castor oil, . . . . . . | gtts. xx. |
| | Carbolic acid, . . . . . . | grs. v. |
| | Tannin, . . . . . . . | ℈ i. *Mix.* |

Several applications are to be made, one after the other, so that a good outer covering is obtained at once. If the patient is weak, give him tr. iron, 20 drops, every four hours; also.

| | | |
|---|---|---|
| ℞. | Fl. ext. Peruvian bark, . . . . | gtts. xx. |
| | Spts. ammon., arom., . . . . . | grs. xx. |
| | Infusion gentian, . . . . . | ℥ i. *Mix.* |

Repeat as often as deemed advisable.

This is the only treatment I have found recommendable in such cases. Psoas abcess, pus in the joints, &c., can be so treated, to great advantage and safety to the patient.

NEURALGIA ACCOMPANYING SHINGLES.—Dr. McCall Anderson asserts, that this form of neuralgia is best relieved by hypodermic injections of morphia.

OPIUM IN ULCERATIONS.—Dr. Anderson, says, opium in small doses frequently repeated is of value in the treatment of affections of the skin, occurring in broken-down subjects, especially in cases of ulceration—doing good, however, more by their stimulating than narcotic properties.

PERTUSSIS CURABLE BY LOCAL TREATMENT.—Dr. W. F. McNutt, San Francisco, Cal., says, (Boston Medical and Surgical Journal,) that his experience is, that most cases of Whooping-Cough can be cured by local treatment, and that one needs only try the treatment to be convinced of the fact. Dr. E. Watson, of Glasgow, recommended a strong solution of Nitrate of Silver to the interior of the larynx, as a successful method of treatment. Dr. G. B. Wood says, referring to inhalations, that the substances used in this way, among others, have been cherry-laurel-water, camphor, tar, benzoin, galbanum, nitrous acid vapors, etc. Children living in the vicinity of gas-works, get well rapidly—which, in these cases, is due probably to inhalation of ammonia, vapor of tar; with the vapor of several volatile oils.

Dr. Caldwell recommends the following formula:

| | |
|---|---|
| ℞.—Ext Belladonna, . . . | ft. gtt. v to x. |
| Potass. bromide, . . . . . . | ℈ i. |
| Ammon. bromide, . . . . . . | ℈ ij. |
| Aqu. distil., . . . . . . | ℥ ij. M. |

S.—Tablespoonful at each application, with atomizer.

Dr. McNutt has always used a solution of Nitrate of Silver, gr. xv. to the ounce, applied by the spray atomizer, and has found the treatment so satisfactory that he has never changed it. One child improved after the second sitting.

ALKALIES IN SKIN DISEASES.—Dr. Anderson, says: Alkaline medicines are specially useful in the treatment of skin diseases, occurring in rheumatic and gouty subjects. He uses the following formula for this purpose:

| | |
|---|---|
| ℞.—Carb. Ammonia, . . . . . . | ℥ i. |
| Solution of Arsenic, . . . . . . | ʒ iij. |
| Syp. Ginger, . . . . . . . | ℥ vi. |
| Infus. Cascarilæ ad., . . . . . . | ℥ xxiv. |

S.—A tablespoonful in half a tumblerful of water, three times a day, after food.

VOLTILE LINIMENT.—An elegant formula is the following:

| | |
|---|---|
| ℞.—Liquor Ammonia, . . . . . | f. ℥ j. |
| Oleum Morrhuæ, . . . . . | f. ℥ ij. |

This a valuable mode of administering Cod Liver Oil by inunction, in chronic and wasting diseases of children, rheumatism, etc.

QUININE PILLS.—The following method of making pills of quinine is furnished by the *Pharmacist*:

| | |
|---|---|
| R.—Sulp. Quinine, . . . . | grs. xxx. |
| Tartaric Acid, . . . . . | grs. iv. |
| Aqua, . . . . . . . | gtt. i.—M. |

Make pills of the required quantity.

Emulsio Hydrocyanata.—Professor Oscar Oldbery says:—Physicians often wish to use hydrocyanic acid, but find it impossible to rely on the article that they are most likely to obtain from the drug-store. The *officinal* preparation is good, but in nine cases out of ten it is not found.

The only way to remedy this evil is for physicians to prescribe some preparation which can conveniently be made "*ex tempore*," and for which no substitute is possible. Such a preparation is the emulsio hydrocyanata, officinal in the Swedish Pharmacopœia, to the exclusion of dilute hydrocyanic acid. It is not new; it was officinally introduced in 1845, and has stood the test. Liebig and Wohler demonstrated that when 17 grains of amygdalin were decomposed by the presence of emulsin, it gave rise to exactly one grain of anhydrous hydrocyanic acid.

This is the formula for the preparation of emulsio hydrocyanata: An emulsion is first made from three parts of sweet almonds, two parts of sugar, and twenty-four parts of water, "secundum artem." To 80 parts of this emulsion is added one part of amygdalin, and the mixture is allowed to macerate one hour. Each ounce of this emulsion contains one-third grain of anhydrous hydrocyanic acid, corresponding to about 32 grains of the dilute of tho U. S. P. Dose:—1 to 2 teaspoonfuls.

The preparation is reliably uniform, elegant, and readily prepared whenever wanted for use.—*National Med. Jour.*

Velpeau's Mixture for Diarrhœa, Dysentery, etc.—The subjoined formula is the original Velpeau Mixture:

℞.—Tinct. Opii.

Tinct. Camph. Comp'd.

Tinct. Rhei., . . . . . . aa. ℥ i.

Ess. menth. pip, . . . . . . ʒ x.

Tinct. Capsici, . . . . . . . ʒ vj.

Misce.

—*Med. Record.*

Treatment of Psoriasis by Balsam of Copiava.—Dr. Henry S. Purdon, Belfast, Ireland, calls attention to the great value of Copaiva in the treatment of Psoriasis. He remarks, that the treatment of Psoriasis, when no acute symptoms were present, by large doses of Copaiva, given with a little liquor potassæ, mucilage and water, has been highly gratifying, especially in cases where it has produced extensive urticaria—indeed the dose should be increased until the latter is established. He has also been able to discharge the patients sooner by the means of the balsam treatment than by any other.

Propylamin in Rheumatism.—Dr. John M. Gaston, in an article in the Indian Journal of Medicine, upon the use of propylamin in rheumatism, says: "Most cases of acute rheumatism are ushered in by chill, fever, and general disturbance, as well as pain. I ususally see that the patient is in a proper condition for the use of the agent, his bowels not constipated. I sometimes order a cathartic, and I frequently premise its use by administering 15 or 20 grains of quinine, in the first twenty-four hours to an adult, after which from 2 to 6 or 8 drops of the liquid propylamin in a tablespoonful of water every two hours for the first twenty-four hours, and at longer intervals the next twenty-four hours, and the cure is accomplished, so far as relief from soreness of the joints and pain is concerned.

The propylamin is found in the shops in two forms, the liquid and the chloride, or muriate. The former is a colorless, transparent liquid, with a singular ammoniacal, and fish-brine odor; is soluble in water, and has an alkaline reaction, and in solution of 2 to 10 drops in a teaspoonful of water is nearly tasteless, and is, so far as I have been able to learn, devoid of poisonous or injurious properties. Its chemical equivalent is $C_6H_9N$.

The chloride is in the form of white crystals, very soluble in water, one grain of which is equivalent in action to about one drop of the liquid.

The agent in either form is somewhat expensive, and that has perhaps been a hindrance to its general use. It formerly sold for five dollars an ounce in this city, but it is cheaper now, costing about three dollars per ounce. I imagine it is sometimes diluted as found in the stores, and if it should fail sometimes on trial, it might be well to bear that in remembrance, and increase the dose. It is said to exist in cod liver oil, in ergot, in chenopodium, and in sorghum, and is extracted chemically from opium and several other sources, but the most abundant source of its supply is found in herring-brine.

A very convenient formula for its administration is as follows:

R.—Propylamin, . . 50 to 80 or 100 drops.
Distilled water, . . . . . . 8 oz.
M. S.—Dose, tablespoonful every two hours to adult.

This is a larger dose than was use by the authority above referred to, but experience has assured me that it is within bounds of perfect safety.

For Inflamed Nipples During Lactation.—A very thick mucilage of gum arabic applied upon the nipples with a feather, or camel's-hair pencil, every time the infant nurses.

PULMONARY TUBERCULOSIS TREATED BY THE USE OF COD LIVER OIL, SAPONIFIED WITH LIME.—Dr. Van Dencosput claims (Kansas City Med. Journal,) for his saponified preparation better results than from any lime, or Cod Liver Oil treatment alone—or indeed, from any other treatment, whatever. His formula is as follows: One hundred grammes of Cod Liver Oil are to be saponified and brought to the consistency of a pill-mass, by the gradual admixture of a sufficient quantity of Caustic Lime. One gramme of the Oil of Bitter Almond is added, and the mass is divided into pieces, of one-fourth of a gramme, (four grains) and covered with some kind of powder. Dose:—Six to eight pieces a day; two after each meal. Under this treament, which does not disturb the stomach, he claims to have frequently seen the diarrhœa arrested or diminished, the evening temperature lowered, the colliquative sweats controlled and the weight of the body greatly increased.

FOR DYSENTERY.—Dr. C. H. Smith, Ellaville, Ga., highly recommends the following formula for Dysentery:

℞.—Chalk Mixture, . . . . . . ℥ viij.
Blue Mass, . . . . . grs. viij.
Tr. Opii, . . . . . . . ʒ ij.—M.

S.—Tablespoonful every 2, 3 or 4 hours, according to circumstances.

Butter-milk to be drunk ad libitum. This treatment does not check the dysenteric discharges suddenly. Dr. Smith rarely fails in his management of the disease by the above.

PHOSPHATE OF LIME IN SICKNESS OF PREGNANCY.—Dr. Medcalfe Johnson, M. R. C. S. E., Lancaster, England, highly praises the hydrated phosphate of lime in the sickness of pregnancy. He has been for some years in the habit of prescribing this medicine, in doses of from three to ten grains, three times a day, suspended in water and flavored according to the taste of patient.

ANTI-HYSTEIC MIXTURE.

℞.—Tinct. Assofœtidæ, . . , . ʒ ii.

Spiritus Ammonæ Aromatic, . . . ʒ ii.

Aquæ Camphoræ, . . . . . ℥ vii.

*Misce et Signa.*—Give one to two tablespoonfuls every three or four hours. P. F.

CARBOLATED CERATE DRESSING IN VARICOSE ULCER OF LEG.—Prof Andrews, of Chicago, (Chicago Medical Examiner,) injects several of the varicose veins with tinct. ferri murias, and dresses the ulcer with antiseptic ointment, composed of carbolic acid cryst., 10 grains, adepis ℥j.

Management of Measles.—Dr. N. S. Davis, of Chicago, Ill., (Chicago Medical Examiner,) considers the following formula one of the best preparations in the first stage of severe cases of measles: R.—Syrup scillæ comp,, ℥ iss; tinct. opii camph., ℥ ij; tinct. verat. viride, ʒ j. M. Dose, one teaspoonful every three hours in a tablespoonful water.

This will, usually, in the course of twenty-four hours, lessen the fever and modify the cough, while the pain in the head will, at the same time, be greatly relieved.

He is not in the habit of giving cathartics until the eruption is fairly out; when, if the bowels have not moved for a couple of days, he directs a mild laxative like the following: R.—Hydrarg. chl. m. gr. v.; leptandrin, gr. ij.; sodæ bicarb. gr. v. M. One important thing to guard against is, extension of the irritation from the bronchial tubes to the lobules of the lungs; making it complicated with lobular pneumonia. When symptoms of pneumonia occur in connection with measles, the best remedy in children is a combination like the following: R.—Liq. ammon. acetas, ℥ iss.; syrup ipecac, ℥ ss.; tinct opii camph., ℥ j.; tinct. verat. viride, ʒ j. Dose—proportioned to the age of the child: for a child two years old, about twenty drops, though it is best to begin with ten drops; for an adult one teaspoonful. It should be given every two, three, or four hours, till the fever is controlled. In the active stage of the disease, while using this mixture, cover the chest externally with fomentations.

Dr. Davis has considerable faith in the popular notion about onions; they certainly afford more relief to the breathing than any other thing. He attributes it to the impregnation of the air which is inhaled, with the volatile oil, more than to any absorption from the surface of the chest, and think this application preferable to blistering.

Wash in Phyalism.

℞.—Creasoti.

Olei Terebinth, . . . . *aa* gtt. xx.

Aquæ Dest., . . . . . . ℥ iv.

Pulv. Acaciæ, . . . . . . q. s.

*Signa.*—Wash the mouth and gums every two or three hours.

P. F.

Sumac Poultice for Boils.—Take the bark of the root of the *white sumac*, and make a poultice with common meal. The bark, after being boiled, can be easily incorporated with the meal. This simple remedy, we are assured, will arrest the progress of boils (even after they have assumed considerable size,) by being continously applied in the form of a poultice, as above directed. Try it.

Permanent Contraction of a Limb Cured by Sub-Cutaneous Injection of Atropia.—M. Destrez obtained a remarkable success in the case of a delicate young lady who had been, for some years, subject to articular rheumatism. A solution of sul. of Atropia was prepared, 1 part in 400—of this, 25 drops (1-15 grain) were injected. Three days after there was marked improvement in the limb. A second injection of 30 drops, (1-12 grain,) and this was followed, at intervals, by three others of 30 drops, which completed the cure.—*Practitioner.*

Anti-rheumatic Liniment.

℞.—Tinct. Opii.
—— Belladonna.
Pulv. Camphoræ.
Aquæ Ammonæ.
Olei Terebinthinæ.
—— Sassafras.
—— Origani, . . . . . . *aa* ℥ ii.
Tinct. Capsici, . . . . . Oi.
Misce.

*Signa.*—Apply to the affected parts twice a day. P. F.

Abortive Treatment of Felons.—Dr. J. H. S., in the Boston Journal of Chemistry, has, for ten years, adopted the plan of applying several coatings of collodion over the finger, or place where the pain is felt, on its first appearance. On drying, the collodion contracts with an even pressure, and if kept on for twenty-four hours the symptoms will, usually, entirely disappear. Of late, he has been in the habit of soaking the finger in quite a strong solution of carbolic acid, for a few minutes before applying the collodion.

Peritonitis Meretricum.—Dr. R. W. Egan, Dublin, (British Medical Journal,) referring to a paper by Mr. Giles, in the same journal, on the association of gonorrhæa with peritonitis, suggests that a not uncommon case of peritonitis among females of that class is found in the prevalence of a practice of interfering with the menstrual discharge just at its inception, or during its course. He has on several occasions attended women for ovaritis and peritonitis, resulting, as he believed, from the application of cold to repress, or the introduction of sponge to conceal, the existence of the catamenia. This latter practice he discovered in making a vaginal examination in a case, and finding what certainly was a foreign body. All those cases were obstinate and slow of recovery.

TREATMENT OF INFANTILE PARALYSIS.—Dr. Brodhurst, of London, (St. Thomas Hospital Reports,) recommends galvanism, as furnishing the most important remedy in the treatment of this disease. The induced current is useless. A galvanic battery of great tention, but of small quantity, is needed—one having not less than fifty cells. There is little hope of recovery if the treatment is delayed six or eight weeks. When the muscles regain sensitiveness to faradization, even in a slight degree, recovery is a mere matter of patience and perseverance. Strynchnia is used hypodermically to the diseased part, in addition to the local treatment by galvanism. He uses a solution of grain of the alkaloid to fifty of fluid. The solution, aided by a little hydrochloric and spirit, is very dense; and if it falls below a temperature of about 70°, the alkaloid crystallizes out. It should always be warmed before use. This solution may be employed with much more freedom than a weaker one. He has freqently injected in quite young children—in fact, in babies—five and even seven minims; that is to say, one-twentieth or one-fourteenth (.05 or .07) of a grain, at a first commencement.

OINTMENT FOR OLD SORES.

℞.—Olei Olinæ.

Sevi.

Terebinthinæ.

Ceræ Flavæ, . . . . . . *aa* ℥ iii.

Cupri Sulphatis.

—— Acetatis, . . . . . . *aa* ℥ i.

Melt the first, second and fourth articles together, and then add the other ingredients and stir until cool. This is one of the best ointments I have ever used on old indolent ulcers and sores.

P. F.

DIAPHORESIS IN SCARLATINAL DROPSY.—The Lancet reports, under the care of Dr. Garrod, "two cases of scarlatinal dropsy, which had been sucessfully treated by hot baths and packing. The patients were first placed in a hot bath, and on leaving it they were, while still wet, enveloped in a hot blanket, and laid in bed beneath one or more other hot blankets, so as to maintain, for a time, a profuse perspiration. The sweating was at first made to last about a couple of hours, but, as the dropsy disappeared, the duration of the process was gradually reduced to half an hour. When all puffiness had disappeared, a treatment by small doses of sulphate of iron was substituted until all trace of albumen in the urine was lost."

A Substitute for the Compound Pill.—A writer in the Chicago Medical Journal suggests the following formula, which he would substitute for the ordinary compound cathartic pill: R.—Aqueous extract of aloes, podophyllin, gamboge, *aa* gr. xxx.; oil of caraway, m. vj. M. Ft. pil. no. lx. He has had the pills made up and sugar-coated in a very satisfactory manner, by J. W. Ehrman, a Chicago pharmaceutist. The pill is small, weighing only three grains after being sugar-coated. One taken at bedtime will usually cause an evacuation from the bowels in the morning, and without occasioning either pain or nausea; but should one fail or be thought insufficient, another may then be taken.

The aqueous extract of aloes seems to contain the more cathartic principles of the drug, while it is the least irritant to the bowels, and the amount administered being so small, it does not appear to act injuriously, even in cases in which the effect of the ordinary dose of aloes is unpleasant.

Anti-Asthmatic Mixture.

| | | |
|---|---|---|
| ℞.—Galbani, | . . . . . . . | ʒ ii. |
| Aceti Scillæ. | | |
| Aquæ Hæniculi, | . . . . | *aa* ℥ iiss. |
| Liquor Ammoniæ Acetis, | . . . | ʒ ii. |
| Spiritus Ætheris Nitrici, | . . . | ʒ i. |
| Syrupus Altheæ, | . . . . | ʒ ss. |

*Misce et Signa.*—Dose three or four teaspoonfuls a day. In humid Asthma this is equal, if not superior, to any remedy that we know of. P. F.

The Iodide of Potassium in the Treatment of Aneurism. At a recent meeting of the Edinburgh Medico-Chirurgical Society, Dr. George W. Balfour exhibited a patient laboring under aneurism of the innominata, whose tumor, from being the size of a small orange, decreased to that of a walnut, under the treatment of rest, iodide of potassium in semi-drachm doses, three times daily, and the external application of iodide ointment.

Pyæmia and its Treatment.—Professor Wood, of London, has lately had under his care, in conjunction with Dr. Beal, two cases of pyæmia—one of the cases is now well and discharged. The treatment consisted of carbolizing the patient—first, by means of keeping the body in a carbolized atmosphere, employ ng small muslin bags filled with McDougall's powder, placed in the bed, and keeping the bed clothes raised by means of a cradle; and secondly, by the internal administration of sulpho-carbolate of iron. The second case is also doing well.

Sesquichloride of Iron and Glycerine in Diphtheria and Croupous Affections.—Prof. Clar (Practitioner, July 1, 1871, from the Wein. Medizin. Zeitung, No. 19, 1871), having experienced the advantages from the application of the best anhydrous glycerine (of Sarg) in various catarrhal and slight croupous and diphtheritic affections, met with some of a much more severe type, which led him to add the sesquichloride of iron to the glycerine with excellent results. The method of treatment he adopts, varying of course with individual peculiarities, is the following: He first prescribes a gentle aperient, either in the form of a manna draught, or of a few grains of calomel, which last he holds to be a powerful antiphlogistic remedy, and, when properly used, of great value. Coincidently he directs cold compresses or cloths to the neck and head, or even to the chest, carefully renovated in accordance with the elevation or depression of the temperature, cold or iced-water being at the same time given as a drink; and then commences at once the use of iron-glycerine, which consists of two ounces of anhydrous glycerine and twenty drops of the liquor ferri sesquichloridi. Of this mixture half a teaspoonful is to be given every half-hour throughout the day and night. As soon as the symptoms appear to be mitigated, the quantity is diminished to a teaspoonful every second hour; and in the intermediate period, with the object of dissolving the exudate, a mixture composed of glycerine, two ounces, borax, twenty grains, is similarly given by a teaspoonful at a time. The iron-glycerine is progressively given at longer periods, and is gradually replaced by the borax-glycerine.

Anti-diarrhœal Mixture.

| | |
|---|---|
| ℞ —Argent. Nitrat. Crystall., . . . . | gr $\frac{1}{4}$. |
| Auqua Distill., . . . . . . | ℥ ii. |
| Pulv. Acac., . . . . . . | ℈ ii. |
| Sacch. Albi, . . . . . . | ʒ ii. |

*Misce et Signa.*—Give one teaspoonful every 2 to 4 hours, in advanced and obstinate cases of diarrhœa occurring in children, especially in newly-weaned infants. P. F.

Bromide of Potassium an Antidote for Strychnine-Poisoning.—In the New York Medical Journal, for March, Dr. Hewlett relates a case of poisoning by five grains of strychnine, which recovered under treatment by bromide of potassium, in doses of ninety grains every half-hour at first, and afterwards in smaller doses at longer intervals. In thirty-six hours from the first dose of bromide, the patient was walking about, feeling a little weak, and having occasionally a slight twitch.

Treatment for Deafness, by Dr. Curtis.—After having cleansed the auditory meatus, and re-opened, so to speak, the orifice of the passage, by removing the morbid secretions which obstruct it, which I think, is best by placing, at night, in the auditory meatus a piece of cotton, moistened with a preparation composed of half an ounce of beef's gall and a drachm (un-gros) of tincture of castor, or tincture of musk. In the morning I syringe the ear with warm water, to which may be added a little soap and a few drops of cologne. I have often substituted, with advantage, for the preparation of beef's gall and tincture of castor, the solution of potass of our pharmacopœia (London,) with the oil of sweet almonds to dissolve the cerumen.

When the ear is well cleansed and the glands are in such a state that stimulant can act upon them, I use the following solution:

℞.—Creosote, . . . . . . . ʒ i.

Oil of Sweet Almonds, . . . . . ʒ iv.

M.—And with a badger's-hair pencil put a small quantity in the auditory passages night and morning. The creosote can be increased according to the effect obtained. Cases, however, present themselves, in which no good result will be obtained from this application without applying, behind the ear, a vesicatory of ointment of tartarized antimony, or other derivations. In otorrhœa, and always when there is pain or inflammation, the creosote is contra-indicated. Its application should cause no pain, or unpleasant sensation, but only an agreeable feeling of warmth.

Imperial Mixture or Drink.

℞ —Potassæ Bitartratis, . . . . ℥ ss.

Aurantii Cort., . . . . . ℥ iv.

Sacchari Albi, . . . . . ℥ iv.

Aquæ Bullientis, . . . . . Oiii.

*Macera per horam in vase leviter clauso et cola.*

*Signa.*—Take a wineglassful, or more, *ad libitum.* This is an excellent cooling laxatiive, diuretic and refrigerant, in febrile diseases, particularly in scarlet fever. In fact, in most cases, this is all that is necessary to give, with the proper attention to the throat.. If the skin is hot and dry, a warm bath every two or three hours, is a charming sedative; it moderates the fever and disposes the patient to a calm sleep. P. F.

Belladonna in Tonsilitis.—A correspondent of the Lancet writes, "in reference to the treatment of acute tonsilitis by belladonna, that he has been in the habit of treating this disease by the administration of two-drop doses of the tincture of belladonna every two hours, with the effect of causing a subsidence of the inflammatory phenomena in from 12 to 24 hours."

CARBUNCLES.—As carbuncles often follow each other in the same patient, anything that promises to arrest them would be gladly tried by the sufferers. Dr. Marcet suggests, in the Lancet, a ready method, provided it be employed as soon as the small vesicle appears on the skin.

He says: "If the carbuncle be allowed to proceed, say, for twelve hours beyond its very first appearance, it will run its usual course; but its progress may be arrested by the early destruction of the vesicle and its contents, by means of the cauterizing action of heat. I have adopted many plans to effect this purpose; but the simplest of all, and one which may be considered as always at hand, is the use of an incandescent lucifer-match. The vesicle is to be merely touched, for a fraction of a second, with the red-hot point from five to seven or eight times in succession, when it assumes a dull-whitish appearance from the coagulation of the albumen it contains. The end of the hot wire may be also used. The pain of the operation is really trifling, and it will save from a week to a fortnight's suffering. I have repeatedly applied this form of actual cautery to myself, and shall not hesitate to do so again if necessary.

"In general, within four or five hours after the operation, the pain from the incipient carbuncle has, in a great measure, disappeared, and there is an end to it. It may happen, however, that the carbuncle, at its origin, is deep under the surface of the skin, when no vesicle appears. I have not been so successful with the use of the actual cautery in these cases as in others; but, probably, had the cauterization been carried deeper, the mischief might have been arrested."

Dr. Marcet has tried nitric acid, and nitrate of silver, but found them unreliable. He thinks the early vesicle may contain a virus, by destroying which, the disease is nipped in the bud. This simple mode is likely to be tried further.

Dr. J. C. Nott, in the New York Medical Journal, for January, records a case which he says is "the only real abortion of a carbuncle he ever saw." It was three inches in diameter, and involved the tissues very deeply. He made a deep incision of one and a-quarter inches, and stuffed it with cotton, saturated with pure carbolic acid, and also painted the whole hardened surface with the remedy. Dr. Nott says: The patient complained of a sharp burning sensation for a few minutes, when the pain subsided completely. The cuticle, by the next day, came off, and the surface looked like a burn. After the first few minutes he was free from pain, and never complained of any afterward. I continued every day, for a week, to insert the acid, in the same way, into the cut, which sloughed all around to the depth of one-eighth of an inch; the surrounding inflammation and induration subsided rapidly, and in a week there was nothing left to treat but the small open wound made by the knife and acid. Three other small carbuncles commenced an inch or two from the large one; they were all treated by incision and the acid, and they all aborted.—*The Doctor.*

Arsensic in Prolapsus Uteri, from Enlarged Cervix.—Dr. L. S. Blackwell reports a case of a patient, aged 48 years, who had been suffering for a number of months from pruritus vulvæ and leucorrhœa—the result of prolapsus uteri.

The intense itching, which was a significant symptom, induced this lady to seek relief. Tournie's treatment—calomel ointment and camphor was resorted to, but failed to afford any *decided* relief; the pruritus, however, gradually diminished, and finally ceased.

With the view of relieving the engorgement and consequent prolapsus and leucorrhœa, the following constitutional and local treatment was instituted:

| | |
|---|---|
| ℞.—Liq. Potassæ Arsenic, . . . | f. ʒ iijss. |
| Syrupi, . . . . . . | f. ℥ j. |
| Aquæ. . . . . . . . | f. ℥ iij.—M. |

A teaspoonful after each meal.

| | |
|---|---|
| ℞.—Iodinii, . . . . . . . | gr. xv. |
| Potassii Iodidi, . . . . . | ʒ j. |
| Aquæ, . . . . . . . | Oj. |

Use as an injection twice a day.

At the expiration of a week, the congestion of the cervix and the leucorrhœa were materially reduced; and at the close of the subsequent week, the engorgement was entirely removed, and the discharge had ceased.

Having used the local application in a number of cases of leucorrhœa, dependent upon engorgement of the cervix, with beneficial results, I have long regarded it as an efficient remedy in the treatment of this exhausting disease.

But never having witnessed such *signal* benefit as was manifested in the case presented, I am disposed to attribute *more value to the constitutional medication.*

Dr. Hunt found arsenic a valuable remedy in diarrhœa; and M. Imbert-Gourbeyre speaks of its "great utility" in the treatment of *vulvar pruritus.*

As sterility and painful menstruation may have their origin in a diminished caliber of the uterine canal from engorgement, it is rational to believe that the efficacy of arsenic depends upon the influence it exerts over uterine engorgements.

The value attached to the general treatment in the case reported, is enhanced by an article entitled "The necessity of associating constitutional medication with topical applications in the treatment of uterine diseases," read before "the Gynæcologocal Society of Boston," and published in the first number of the Gynæcological Journal. Arsenic has also been used with satisfactory results in treatment of hæmmorrhoids, and from the efficacy which has been attributed to it in the therapeutics of the pelvic viscera, we are convinced of its special affinity and adaptation to morbid action in this section of the human organism.—*Med. & Sur. Reporter.*

Carbolic Acid.—Dr. C. F. J. Lehlback, says: "In various species of Impetigo, and particularly of porrigo, in tinea favosa, there is no local remedy in my experience attended with better and more speedy results. In an inveterate case *tinea favosa*, which occurred in a patient fifteen months of age, during dentition, having existed for nearly a year, resisting various modes of treatment, reducing the child by its irritative effect to a minimum of vitality, depriving it of sleep and interfering with the digestive and assimilative processes, and which had been pronounced by several physicians, advocates of *humeral* (*humerous!*) *pathology*, as beneficial, (because, to cure the external disease, would drive it *inward*,) carbolic acid was used for three weeks, and a permanent cure effected. The mode of using the remedy was in form of ointment, as follows:

| | |
|---|---|
| ℞.—Acidi Carbolici, Crystal, . . . | dr. one. |
| Sodæ Sulphitis, . . . . . | dr. half. |
| Glycerine, . . . . | oz. one and a-half. |
| Cerati Simplicis, . . . . . . | oz. two—M. |

S.—Ointment to be used three times a day.

The benefit derived from this application may have been owing partly to the sulphite of soda. But in other incipient cases of this class I have used carbolic acid alone, (acid. carbol. cryst. gr. xx., glycerinæ ounces, ss, cerat. simpl. ounces, i,) and a few days' application proved sufficient to annihale the trouble.

In Scabies, I have employed it with decided success, (better success than with sulphur preparations alone) in conjunction with the letter. The following is a good ointment:

| | |
|---|---|
| R.—Acid. Carbol. Crystai., . | oz. one and a-half. |
| Flor. Sulphur. . . . . . | oz. half. |
| Sodæ Sulphit., . . . . . . | oz. half. |
| Glycerine, . . . . . . | oz. two. |
| Cerat. Simpl., . . . . . . | oz. two.—M. |

S. Ointment. Take a bath, or wash all over, morning and night, and then anoint thoroughly wherever the disease exists. The acari are generally killed in three to five days.

SULPHITE OF SODA IN CARBUNCLE.—Dr. S. J. Radcliffe, (Med. Sur. Reporter,) says: "In a case of carbuncle that recently came under my observation, of enormous size, and fast undermining the constitution, after using the ordinary treatment, including the knife, with but indifferent results, resort was had to sulphite of soda, and the condition of the general and local symptoms were changed in a marked degree, almost from the commencement of its use, and the recovery was speedy and complete. No case have I ever treated with more satisfaction to myself or the patient. There is, I believe, much encouragement to be looked for in the treatment of all such diseases with these remedies and I think good results may confidently be expected in their exhibition at all times.

From 1 to 2 drachms may be given daily.

In external exhibition, I use it in simple solution in water, or glycerine and water combined. In ulcerations of the mouth and throat, I directed a simple solution, as above, as a wash or gargle; and as a wash in syphilitic sychosis it acts with remarkable promptness.

HOOPING-COUGH.—In the treatment of hooping-cough, Dr. Jas. P. McVicker, has derived the most signal advantage from the exhibition of the fluid extract of cimicifuga, in doses suited to the age of the child, and repeated every hour: and the same gentleman has prescribed the bromide of potassium in the eclampsia of children with the most satisfactorv results.—*Trans. Pa. Med. Soc*

FORMULA EOR GIVING QUINIA.—A correspondent furnishes the following formula, intended to cover the bitter taste of quinia:

| | |
|---|---|
| R.—Quiniæ sulphatis, | gr. xxiv. |
| Syrup sarsap. comp., | f. oz. iij. |
| Acidi tannici, | gr. iij. |
| Ol. Menthæ, | gtt. v. |

—*Medical and Surgical Reporter.*

COLLINS' DISINFECTING POWDER FOR FOUL APARTMENTS.—Two parts of fresco and dry chloride of lime and one part of burnt alum intimately mixed, are found more efficacious in destroying or neutralizing putrid ordors than either of these substances when used alone. The compound powder may be placed on a shallow plate or similar article; if the weather be damp, no addition of water is necessary, but in a very dry atmosphere, a little water is better.

---

GEORGE C. REYNOLDS, M. D., a leading man in one of the mission schools of Chicago, goes out soon to Eastern Turkey as a missionary of the American Board.

TREATMENT OF GONORRHŒA.—Mr. Berkley Hill (Lancet, April 29, 1871) speaks favorably of the jelly of copaiba in the treatment of the sub-acute form of this disease. This preparation is almost as firm as calf's-foot jelly, very attractive to the eye by its rosy red color, and not repulsive to the palate, its flavor being masked with peppermint. As it contains seventy-five per cent. of copaiba, large quantities can be taken in a small bulk. If a piece as large as a filbert be rolled in wafer paper, it can be swallowed without being tasted at all. The after-effects of nausea, diarrhœa, etc., are not more frequent than from other forms of copaiba, if indeed they be as common. Mr. Hill speaks favorably of the oil of sandalwood, but does not think it superior to copaiba. The following formula of Henderson is reccommended in conditions not permitting the use of copaiba: Oil of sandalwood, one ounce; rectified spirits of wine, two ounces; oil of cinnamon, twenty-five minims. Dose, one or two drachms three times a day.

CHRONIC ULCERS—Dr. J. B. Mobley extolls the following treatment for Chronic Ulcers:

R.—Expressed juice of Passiflora Incarnate, Oss.
Ol. jec. Ascelli, . . . . dr. iv.—Mix.
S.—Apply thrice daily.

He has used this application in four inveterate cases with perfect success.

QUININE BISCUITS.—One of the London bakers has introduced a dietetic novelty in the shape of quinine biscuits. They are small, extremely well made, and have a pleasant and delicately bitter flavor, not too strongly pronounced, which is exactly what a club man seeks in his sherry and bitters. Each biscuit is estimated to contain one-fourth of a grain of quinine, and for delicate stomachs, or where it is desirab.e to disguise medicine as much as possible, or to combine food with medicine in a perfectly agreeable from, these biscuits are likely to become very popular.—*English Journal.*

MANAGEMENT OF INCONTINENCE OF URINE.—John Barclay, M. D., of Aberbeen (Med. Times and Gazette), during the past two years and a half has successfully treated twenty cases of incontinence of urine by the syrup of the iodide of iron alone. The improvement in several of the cases followed so closely on the administration of the remedy as to leave no doubt but that the good effect was due to the syrup. Dr. Manson, of Baniff, and Dr. Smith, of Kinnairdy, have both found the medicine equally satisfactory.

Gonorrhœal Peritonitis.—A correspondent of the British Med. Journal calls attention to gonorrhœa as a cause of peritonitis in the female, and believes the Fallopian tubes to be the avenues through which the inflammation reaches the peritonæum. In his experience, the disease differs from puerperal peritonitis in being less severe and of shorter duration, in manifesting improvement upon the removal of as much of the cause as is possible by means of injections of warm water, and upon treating the inflammation by the usual means. No case, he says, has proved fatal in his practice; but he fails to state the number which he has had under observation.

Torticollis.—New Method of Operating.—Prof. Wood, of King's College Hospital (Med. Press and Circular), has devised a new operation for wry-neck, instead of the subcutaneous tenotomy of the *origins* of the affected sterno-cleido-mastoideus muscle. It is the substitution of the subcutaneous tenotomy of the *insertion* of the muscle at the mastoid process, instead of the former operation, which is generally practiced. In reporting a case, the Professor stated that it was the *third* of the kind on which he had successfully operated, by substituting division of the *insertion* of the sternomastoid muscle, in preference to its origin.

To Prevent Infantile Gonorrhœal Ophthalmia.—When the lying-in woman has gonorrhœa, Dr. Brandeis, of Louisville, (Rich. and Louis. Med. Jour.,) would suggest that the child be allowed to be born, if possible, without a rupture of the membranes, thus protecting the eyes of the infant against this cause of ophthalmia.

Stricture of the Urethra.—A reviewer in the Med. Press and Circular, in speaking of the great reputation gained by Mr. Holt, of Westminister Hospital, London, says that there cannot be any doubt that his dilator is the very instrument invented by Perreve many years ago in Paris. Mr. J. D. Hill, surgeon to the Royal Free Hospital of London, has recently added to the fame of Mr. Holt's operation by relating the history of 120 cases of stricture thus treated. Of these 120 patients operated on by Mr. Hill, by Holt's method, 118 recovered, and there were two deaths; and of these 118, all except one could have No. 11 passed before leaving the wards.

In cases attended with complications, Mr. Hill, before using Holt's dilator, advocates the washing out of the bladder by means of a lotion composed of twenty drops of strong nitric acid in a pint of water, to which half an ounce of liquor opii sedativus is added.

This surgeon thinks that Holt's operation is the most satisfactory method of treating any form of organic urethral stricture which is amenable to dilatation, and that, with careful attention to preliminary treatment, there is no more risk to run in its employment than in ordinary dilatation. He affirms that the rate of mortality is probably less than in gradual dilatation, when it is considered that a certain proportion of patients die from extravasation of urine, and from complications following ordinary catheterism.

SULPHITE OF SODA IN DIABETES.—Dr. C. B. Kieffer, Carlisle, reports two cases of Diabetes cured by the use of Sulphite of Soda., In one case he gave the following fomula:

R.—Sodæ Sulsphitis, . . . ½ ounce.

Aqua, . . . . . 6 ounces.—M.

S.—A desertspoonful, four times a day, in a little water.

A NEW PREPARATION OF PEPSINE.—Emil Scheffer, pharmaceutical chemist, of Louisville, Ky., (Rich. and Louis. Med. Journal,) asserts that no effects can be expected from a solution of pepsine in a solvent containing alcohol. Neither has he much faith in the dry pepsine, owing to its being mixed with half starch, which is apt to turn musty by the attraction of moisture. The gastric juice containg hydrochloric acid, induced Mr. Scheffer to adopt the more rational plan of preparing his liquid pepsine by using that acid and glycerine. The glycerine in this case not only acts as a preservative, but undoubtedly also produces a soothing effect on the irritated mucous membrane of the stomach. The dose is from one to two teaspoonfuls, after each meal, in cases of dyspepsia, indigestion, and vomiting of pregnant women.

Dr. E. S. Gaillard, of Louisville, adds, that this chemist is worthy of entire confidence, and his preparation is the most valuable that he has used. It has also given entire satisfaction to many of the Louisville physicians who have prescribed it.

TINCTURE ERIGERON CANADENSE IN HEMORRHAGE.—Dr. J. F. Garretson, says: "In any ordinary hemorrhage, where something besides a local means seems necessary for its arrestation, 'Tinct. Erigeron Can.,' given in single drop doses each minute, will be found very reliable. To give it in larger quantities than this, or more frequently, seems to defeat the end. In epistaxis, or the internal hemorrhage, if not too severe, it is very useful, and seldom fails. The erigeron grown in Rhode Island, near the sea-coast, seems to possess the most virtues."

SULPHITES IN SYLPHILIS.—Dr. S. J. Radcliffe, of Washington, D. C., (Med. & Surg. Reporter,) says: "I begin the treatment of Sylphilis with these medicaments, (Sulphite,) and in no case have failed to derive benefit—especially in those cases which had become, so to speak, fixed in the constitution and so hard to eradicate, with sycosis, extensive ulcerations of the throat and various portions of the body, suppurating bobues, etc., etc. Many of the cases had resisted other methods of treatment. He did not confine the employment of the Sulphites to secondary and tertiary Syphilis, but give them in the primary stage, after a slight mercurial treatment. He does not advise their use to the exclusion of all other treatment, but a trial will repay the practitioner. He prefers the Sulphite of Soda, and of this gives from dr. one to dr. one and a-half, in divided doses, 3 or 4 times daily, in simple solution in water, continuously, for a length of time.

ELIXIR CHLOROFORM.—Useful in colic:

R.—Chloroform,
Tinct. Opii,
Tinct. Camphoris,
Spir. Ammon. Aro, . aa. oz. iss.
Oil Cinnamon, . . gtt. xx.
Brandy, . . . . oz. ij.—M.

Sig.—Half a fluid drachm, more or less.—*American Eclectic Medical Journal.*

FALLING OF THE HAIR.—The most satisfactory treatment is the use of Tannin, one and a-half scruples, Adipsis, one oz. Oleum Sabinæ, gtt. v—xxx to one oz. Adipsis, has this effect still more decidedly, but causes the hair to become harsh, stiff, and of a dirty color, and has an unpleasant odor. It sometimes causes severe headache.

CAPSICUM IN ASTHMA.—Asthma, like whooping-cough, has an endless numberof remedies, in and out of the Profession, each of which is pronounced almost a specific. As illustrative of this, we give the following, from the pen of Dr. H. C. Barnard: "I now use *stimulants*, and my favorite is *Capsicum*. This I give, both during the paroxysm and *ad interim*, as a propholactic, and I now have patients who confidently resort to its use whenever they feel any premonitory symtoms of a recurrence of the disease, with almost never-failing success. I say *almost*, for one of my patients has had two attacks, in spite of treatment, within three years, whereas he fomerly suffered as many without.—*Medical and Surgical Reporter.*

Medical Treatment of Ulcer of the Leg.—Dr. Heath relies upon opium, iodide potassium, and arsenic, in the medical treatment of these ulcers ; and thinks each has its appropriate class of cases. He gives opium in the chronic callus ulcer ; in the small irritable ulcer, often found in connection with varicose viens ; in sloughing ulcers, combined with local poulticing. The iodide of potassium he regards as infallible, given in appropriate doses, in syphilitic ulcers. Local mercurial treatment, in the form of yellow or black wash, or the white precipitate ointment, appears to hasten the healing of these ulcers. The iodide of potassium should be given in 10-grain doses, three times a day, increased to 15 or 30 grains, and should be taken for some time after the apparent cure.

Arsenic is very valuable in those cases in which eczema is a concomitant, if not the anticedent of the ulcer. In cases where the skin is irritable, inflamed, and epidermis dry and scaly, the arsenic will rapidly improve and the ulcer heal under any simple stimulant. He thinks the dose should, if necessary, be gradually increased to 10 to 15 minims, and to maintain this high rate until the constitutional symptoms of the drug are produced. The arsenic must always be given on a full stomach, and is an invaluable tonic.—*Lancet.*—*Braithwait's Retrospect.*

*A Good Precsrciption in Incipient Tuberculosis,* prepared as follows :

R.—Glycerine, . . . oz. iv.
Syr. of the Iodide of Iron, . . oz. i.—M.

S.—Give one teaspoonful three to four times a day. It has been used also, with the most happy effects in more advanced stages of the disease.

Treatment of Bronchorrœa by Tar-Water.—The *Eau de Goudrou* which a chemist, in Paris, by the name of Guyot, is advertising and selling largely, is little else than tar-water—aqua picis. It is found of great efficacy in the treatment of bronchorrœa and allied complaints. It is strange that the balsams are so much neglected by physicians. Not long since, one wrote to us that he had been cured of what he believed to be, and what his medical friends pronounced to be consumption, by their use. He seemed to think he had left the regular practice in submitting to this treatment, when, in fact, their use has been recognized as efficacious for generations. They should be more extensively employed.

On the Treatment of Buboes and their Sinuses.—Dr. J. Lampsey writes that a bubo which, under the old method of treatment, might take months to heal, may be cured in a few days by a more judicious mode. The first incision should be made in the centre, in a *vertical* direction, always taking care that the bubo has well suppurated, beforehand.

The next day following the incision, he injects by means of a common glass syringe, a solution of carbolic acid, diluted one to ten, into the bubo. It cause some pain at first, but it soon passes off. This injection should be squeezed out of the sore by moderate pressure, and need not remain in it longer than a few seconds. Care must be taken not to allow the solution to come in contact with the scrotum or the skin, as it sometimes excoriates. He repeats the injection after an interval of three days, applying, in the meantime, over the bubo, a piece of lint wet with carbolic acid, diluted 1–40, and covered with gutta percha tissue.

Should another bubo form in the vicinity, and a communicating sinus be established, he introduces a probe to the furtherest limit of the sinus or bubo sac, and makes a small opening in the integuments so as to allow the extremity of the probe to pass through, and a free current of carbolic acid solution is injected through. In a few days the healing process is satisfactorily established."—*Medical Press and Circular.*

A Good Prescription in Infantile Pneumonia:

℞.—Agnan Dist., . . . . oz. iv.
Pulv. Acaciæ, . . . . . dr. i.
Tinct. Aconite, . . . gtts. v to x.
Tinct. of Ipecac, . . gtts. xx to xl.—M.

S.—Give one teaspoonful every one to two hours—we have used this prescription with great success for many years. P. F.

Glycerine with Tannic Acid in the treatment of Vaginitis, prepared as follows:

℞.—Glycerine, . . . oz. ii.
Tannic Acid, . . . . dr. ii.—M.

The speculum having been introduced, an injection of water is applied in order to remove all the purulent mucus adhering to the walls of the vagina; these afterwards are wiped with a dossil of dry charpie, placed at the end of a long forcep. Introduce then one or more tampons of cotton, soaked in the tannic glycerine, and in addition to it, a dry tampon to retain the drops which tend to escape.

ELECTRICITY IN NEURALGIA.—It has been long known to the Profession that if an intense current of electricity be applied to the course of a nerve its sensory power is benumb for a time, and the same result follows if the nerve is the seat of neuralgic pain. After a time the pain most frequently returns, but on repeating the electrization, the recurrence is at longer intervals, and very frequently a permanent cure effected. We have known of a few and heard of many cases of Sciatica, lumbar, crural and trifacial neuralgia successfully treatted by electricity.

TINCTURE OF ERIGERON AS A HEMOSTATIC.—A writer in the *Dental Cosmos*, recommends tincture of Erigeron as more efficacious than Monsels Salt in arresting the hemorrhage following the extraction of teeth. We suppose the E. Canadense is the species employed—one of the most common and widely diffused plants in America.—*Richmond and Louisville Medical Journal.*

ON THE TREATMENT OF SYLPHILIS.—At the conclusion of a paper on this subject, (*Lancet*,) Dr. Spender recapitulates the therapeutic propositions advanced by him :

1. In secondary Syphilis—iodide potassium in moderate doses, with blue pill, or some analagous preparation.
2. Syphilitic inflamation of deeper tissues—iodide potassium and bichloride of mercury.
3. For intermediate squmous syphilides—bichloride of mercury ; green iodide of mercury ; and in doubtful cases, arsenic with bichloride of mercury.
4. Early tertiary phenomena—iodide of potassium and bichloride of mercury. Later ones—large dose of iodide of potassium. Cures may be effected. Tonics are useful as subordinate agents.
5. Infantile Syphilis—always requires mercury.—*Braithwaite's Retrospect.*

ICE IN AFFECTIONS OF THE TESTICLES.—Diday (*The Annales*) emyloys ice in some of the affections of the testicular apparatus : 1st. In orchitis, sometimes complicated by blennorrhagic epididymitis, he finds it serviceable ; 2d. In testicular neuralgia ; 3d. In certain states in which pain constitutes the predominant feature.

*Sulphate Quinine in Erysipelas.*—Dr. J. C. White, (Boston Med. & Sur. Journal,) thinks that quinine, administered in moderate and fractional doses, promptly and completely arrests the course of non-traumatic erysipelas of the face, and extinguishes it most frequently on the second or third day of its employment.

Treatment of Functional Indigestion.—Wm. Aitken, M. D., Edinburgh, gives the following:

R.—Sodæ Bicarbonatis, . . . gr. xv.
Potassæ Nitratis, . . . . gr. iij.—M.

For one powder, to be taken two or three times a day, in those forms of indigestion marked by excessive acidity and heart-burn. At the same time free action from the liver and bowels must be sustained by occasional doses of blue pill or podophyllin, combined with extract of Colocynth and of henbane while exercise and diet are duly attended to.

R.—Ammoniæ Carbonatis, . . . gr. i.
Ext. Gentianæ, . . . . gr. ij.—M.

For one pill, ter die, in weakened digestion from over-fatigue:

R.—Ext. Nucis Vomicæ,
Ferri Sulphatis, . . . aa. gr. ½.
Ext. Colocynthidis Comp., . . gr. iv.—M.

This combination taken early in the morning generally induces gentle action of the bowels. In prescribing the mineral acids, our author calls attention to the following general rule, stated by Dr. Bence Jones, namely: That the influence of sulphuric acid is astringent, while that of hydrochloric acid promotes digestion, and nitric acid, secretions.

Thomas King Chambers, M. D.—

R.—Acidi Hydrocyanici, diluti, . m. iv.
Infusi Gentianæ, . . . f. dr. ss.

For one dose, ter die, in heart-burn, due to over-sensitiveness:

R.—Zinci Oxidi,
Pil. Aloes et Myrrhæ, . . aa. dr. iss.—M.

Div. into xx pills; one, ter die, in the nervous trembling, indigestion of food, and vomiting arising from indulgence in spirit-drinking, between meals, and in the forenoon.

J. M. Da Costa, M. D.—

R.—Acidi Nitro-muriatici, . . f. dr. ij.
Vini Pepsini, . . . . . f. oz. iij.

A teaspoonful three times a day, before or after each meal. In functional indigestion, owing to want of proper secretion of gastric juice. When there is constipation, add, also:

R.—Pulv. Rhei, . . . . scru. i.
Quin. Sulphatis, . . . . . gr. x.—M.

Divide into x pills, one to be taken at night. If this be not sufficient to produce a laxative effect, take one, night and morning. Meat diet almost exclusively, avoiding starchy substances.

THOS. HAWKES TANNER, M. D.—

R.—Acidi Nitro-muriatici, dilute, . f. dr. ii.
Acidi Hydrocyanici, diluti, . . m. xxv.
Tincture Arnicæ, . . . f. dr. j.
Tincture Gentiana Compos., . . f. dr. j.
Infusi Sennæ, q.s., . . f. oz. iij.—M.

A tablespoonful two or three times daily, in dyspepsia with sluggish action of the liver. The efficacy of this prescription may often be increased by giving, with each dose, the following pill:

R.—Quin. Sulphatis, . . . gr. xij.
Pulv. Ipecac, . . . gr. xij–xxiv.
Ext. Gentianæ, . . . gr. xxiv.—M.

Divide into xij pills, and order one to be taken every day, at dinner. An excellent remedy in slow digestion:

R.—Ferri Redacti, . . gr. xxxvi–dr. i.
Pepsine, . . . . . gr. xxxvi.
Zinci Phosphatis, . . . gr. xviij.
Glycerine, . . . . . q. s.—M.

Divide into xxiv pills, silver them, and order two to be taken every day, at dinner.—*Therapeutical Bulletin*, (*Modern Therapeutics.*)

NITRATE OF LEAD IN ONYCHIA.—Dr. DeMoorloose speaks highly of this salt applied to the sanious fungus ulcers resulting from Onychia. One application of the powdered nitrate every 24 hours is sufficient. After the first dressing the pain ceases, the swelling abates, suppuration is lessened, and the fetid odor destroyed. Any horny or irregular fragments should be removed by excission. The worst cases are often cured in eight or ten days. It might also, be used in scrofulous and unhealthy ulcers. —*Glasgow Medical Journal.*

*Sulpho-Carbolate of Zinc.*—Dr. Jno. Wood was the first to use this new remedy. It is chiefly employed as an external application in the form of lotion. Three to ten grains should be dissolved in an ounce of distilled water. The effect on suppurating surfaces is marked in the arrest of pus-formation and the abolition of all fœtor. Carbolic acid is evolved when thus applied, and is greatly superior to the acid on account of the absence of irritating qualities and all unpleasant odor. Used in gonorrhæa, the Sulpho-Carbolate of Zinc, at once checks the discharge.

MUSCULAR RHEUMATISM.—A correspondent of the *Chicago Medical Examiner* writes as follows about this disease:

"Many years ago, Mr. Carmichael, the celebrated surgeon of Dublin, had sciatica gradually creep on him till he was entirely disabled. For some little time, if I mistake not, before he yielded to it, he had to be carried from his carriage to his lecture-room. He had been in the habit of working very hard in his profession, eight hours a day, from breakfast to dinner, without any form of lunch. This proved too much for him, as it probably will for any man.

"When he concluded to be treated efficiently, he came to a hot spring in the Pyrenees, and had a warm bath every day, with a dose of Pil. hydrarg. every night, and seidlitz powder in the morning. This course restored him the use of his limb within a reasonable time. I have tried a similar course with the best effect in old, confirmed cases.

"But the remedies I use in acute cases are calomel in small doses, 1 or 2 grains, with gum guaiac and Dover's powder, three times a day. The Dover's powder in doses sufficient to soothe the pain, 3 to 5 grs., and the guaiac in 8 or 10 gr. doses. These I accompany with a warm water (with salt) sponge-bath every morning, to be followed by good rubbing, with dry towels. A cathartic of castor oil once in two or three days, if the powders do not act sufficiently, and sometimes if they do act some, is very useful.

"With this course my cases have uniformly yielded. It is quite possible that belladonna would prove a better remedy in these cases.

"In my case, the pain commenced at the sacroiliac junction, and extended across the back affecting the lumbar muscles. It was attended with a general failure of the secretions, gradual whitening of the tongue, and increased heat of the skin. The remedies gave me relief in a very few days."

CHOLERA MORBUS.—A correspondent writes to us as follows: "I am now, and have for the past two years, been treating cholera morbus, cholera infantum and colic, also, the various forms of diarrhœa in adults and children, attended with pain, with salt-water injections, with the best of success. I use but very little medicine in the above mentioned diseases, and in the majority of the cases none at all.

OVARIAN NEURALGIA.—Dr. Francis E. Anstie, states that Ovarian Neuralgia, and painful menstruation, with hysterical depression of spirits, is most effectually relieved by small subcutaneous injections of morphia, or still better, by atropia.—*Practitioner.*

Permanganate of Potash.—This salt has been tried carefully in the Jacksonville Surgical Infirmary, and it is reported that from comparative trials made with it and carbolic acid in erysipelas, putrefaction and complications of wounds, permanganate is believed to be greatly superior to carbolic acid and to everything else which has been tried. It is applied over wounds and upon erysipelatous surfaces in nearly a saturated solution. Over wounds it is best applied by laying on a portion of lint saturated with the solution. This is probably the best application to make to bullet wounds and those attendant upon compound fractures. By preventing the putrefactive change in the exudations and effusions, the extension into them of the organizing process is greatly favored. Care should be taken not to apply the solid salt, as it acts as a caustic.

*Hypoposphite of Potass., in Chronic Bronchitis.*—Dr. Jno. C. Thorowood, in a paper upon the "medicinal use of phosphorus and its compounds," speak favorably of the hypophosphite of potash in chronic bronchitis. In the case of a patient under his care suffering with obstinate chronic bronchitis, with thick fetid expectoration and tendency to congestion of the lungs, 5 grains of the hypophosphite of potash in camphor water, effected a complete cure without any other medication whatever. To test the effect of the treatment, he stopped the medicine during one week, and the effect was a marked increase of cough and thick fœtid expectoration; on returning to the mixture these symptoms were entirely removed and the patient was dismissed, cured. In other cases of chronic bronchitis, remaining after the subsidence of an acute attack of the disease, and not, as a rule, complicated by the presence of emphysema of the lung, he has given the hypophosphite of potash in camphor water, with much advantage. The diagnosis, between chronic bronchitis and phthisis, should be accurately determined, as great mischief results from its incautious administration to persons affected with tubercular deposit in the lungs—causing rapid softening, and in many instances, speedy death.

*Turpentine in Puerperal Prostration.*—Edward Garraway, Esq., recommends turpentine as a restorative in certain states of prostration, especially such as occasionally arise during the puerperal state, as very serviceable. Sometimes, after a severe labor, accompanied or not with hemorrhage, great debility will ensue about the third day, characterised by a rapid pulse, tympanitic abdomen, and other symptoms not connected with peritoneal or other fever, yet threatening the advent of a typhoid condition. Here, turpentine, both as an injection and by mouth, is invaluable.

*Sulpho-Carbolate of Sodium.*—Dr. A. E. Sansom, (Practitioner) remarks that, in his "experience of the use of sulpho-carbolate of sodium in *zymodic diseases* is unexceptionably favorable. During twelve months I have administered it in every severe case which has fallen under my observation. I have not had one fatal case." He employed it in five cases of severe tonsillar ulceration, all of which rapidly recovered without the occurrence of suppuration. In a case of diptheria, with abundant false membrane, he administered the sulpho-carbolate of potassium. Perfect recovery took place. In scarlet fever he used the sulpho-carbolate of sodium. Recovery was, in every instance, perfect, rapid and uncomplicated. All the cases recovered in a less period of time than under any treatment he had previously employed in similar cases. He has used it, with marked advantage, in erysipelas of the face, and in variola. He has given the carbolates in about 200 cases. The dose of the sulpho-carbolate of sodium, is from 20 to 60 grains. It is freely soluable in water, has scarcely any objectionable taste, and children take the salt readily.

TONKA BEAN IN PERTUSSIS.—Dr. John Cooper, of Philadelphia, Pa., has successfully treated hooping-cough, by giving the Fluid Extract of Tonka Bean. He begins with 5 drops in sweetened water, every three hours, increased to 8 drops after the third dose. Relief is soon felt; the paroxysms gradually subside, insuring rest and sleep at night, and the cough assumes more that of ordinary cough than that of pertussis.

USES OF ARSENIC.—We notice, in some of our exchanges, that arsenic in small doses has been recently employed with very satisfactory results in the treatment of hemorrhoids. It was also observed to act very kindly in cases of chronic prostatis, in at least one instance, curing an obstinate and distressing case of many years' standing.

STRYCHNIA AND XANTHOXYLIN IN DYSPEPSIA.—We are informed that some of the most troublesome cases of indigestion have been greatly benefitted by one-thirtieth portion of a grain of strychnia and one grain of Xanthoxylin, taken after each meal.

*Bebeerina in Uterine Diseases.*—Dr. A. P. Merrill (American Practitioner) thinks bebeerina exerts a specific action upon the uterine organs equal to that of mercury on the liver. He has tested its power for years in controlling hyperæmia and morbid hypertrophy of the organ, as well as cartarrhal discharges and morbid secretions of the uterine cavity, the cervix and vagina.

*Strychnia in Epilepsy.*—There are certain forms of epilepsy in which anæmia and defective nervous centre are prominent symptoms. The majority of these patients are women. The disease commences about the time of the first menstruation, and the attack occurs generally at each catamenial epoch. In these cases strychnia in moderate doses, are very serviceable. Dr. Tyrrell has used the remedy for ten years, and deems it valuable.—*British Medical Journal.*

*Cancer.*—Dr. Needham advises the use of the fluid extract of clover in cancer—thinks he has cured himself with the remedy.—*Journal of Materia Medica.*

*Flexible Collodion in Herpes Zoster.*—The Editor of the Dublin Quarterly Journal states that in numerous cases, he found the greatest advantage to result from the immediate painting the vesicated patches with collodion and repeating the same as often as required. He warmly recommends it even in cases where superficial ulceration has taken place. The Styptic Colloid, we think, would be equally, if not more effectual, in these cases.

*Subcutaneous Injection of Morphia in Dyspepsia.*—Dr. T. C. Allbutt, employs morphia, hypodermically, in certain forms of Dyspepsia, with the greatest advantage—curing the disease rapidly. He uses it in "Atonic Dyspepsia"—where there is foul breath, thirst and loaded tongue, depending more upon a nervous condition than upon "sluggish liver," or deranged secretions. This form of dyspepsia often occurs in hysterical women. His success should induce others to try it.—*Practitioner.*

*Sulphurous Acid in Chronic Vomiting.*—Dr. Chas. R. Drysdale, in one case of obstinate chronic vomitting, after repeated failures with bismuth, hyrocyanic acid, etc., promptly relieved the patient by giving half-drachm doses of sulphurous acid in one ounce of water, three times a day.—*Lancet.*

*Subcutaneous Injections of Morphia and Aconite in Convulsions After Labor.*—Dr. R. M. Bowstead, after trying various remedies in puerperal convulsions, regards the hypodermic injection of morphia and aconite as valuable. In one case he injected 2 minims of aconite (Fleming's) and one-third of a grain of acetate of morphia. The convulsions ceased in less than five minutes, and the patient passed into a quiet sleep. In three days she was convalescent.

### RICE—ITS USE.

1. As an article of food the grain is used in almost every possible form—whole, broken, half-broken, coarsely crushed, reduced to powder, or dissolved. Breads of various kinds, and also cakes of delicate flavor, are made of it, combined with the flour of other cereals or used by itself. It is also prepared in the form of pillaus, pies, puddings, custards, soups,, and gravies.

2. As a diet for invalids or for infants, it may be pulverized in a mortar after a few minutes soaking in cold water; or it may be ground in a mill and prepared like sago, arrowroot, or tapioca; or, without pulverizing, it may be made into a light nutritious gruel by boiling and straining.

3. As a medicine, in relieving a disordered condition of the stomach and bowels, it is parched brown, then boiled and eaten; or it is parched of a darker brown, drawn as coffee or tea, and used as a beverage.

4. In the laundry, where a white pearly starch is required, capable of various degrees of stiffness, it is highly valuable.

5. In the manufactures its paste is moulded into a variety of tasteful ornaments, almost as hard as porcelain.

6. Rice meal, the detritus of chaff and grain already described, is a highly nutritious and fattening food for all kinds of stock, from horses to pigs.

7. The rough rice, or paddy, is the favorite food of the rice-bird, so highly prized by epicures, and is probably the source of its peculiarly delicate flavor. It may be given to poultry with similar effects, and it contains so much silex in its rough husk that while feeding on it they need no gravel for attrition in the gizzard. It is therefore peculiarly suited for poultry on long voyages, or in places where there is no gravel in the soil.

8. The chaff is in demand for packing delicate articles of glass or china ware, and for the transportation of eggs. It is at the same time so perfect a non-conductor of heat that it is said a block of ice properly packed in it will withstand the direct rays of a sun hot enough to open the seams of a vessel's deck.

9. The straw is manufactured into beautiful hats and bonnets, and also into mats and light baskets, and like the stalk of most other cereals, is used as a rough provender for cattle.

### IN THE CUISINE.

A lady, familiar from childhood with the luxuries of a rice plantation, furnishes by request the following instructions:

1. To boil rice for the table a la Carolina. First pick it free from rough grains and foreign substances; then wash it qucikly in cold water, and hurry it to the pot before the grain is at all softened. As to the quantity of water to be used in boiling, there are two modes: one is to put in twice as much water as rice, and allow it all to be absorbed by the grain; the other is to put in three or four times as much water, but to pour almost all of it off as soon as the grain changes from its pearly white color and gives proof of having softened.* In both modes, when this stage of the boiling has been attained, the pot is to be withdrawn from the hot fire ane set where it will be kept at a low steaming heat, until the water is all gone. This last process is called "soaking." Properly conducted the rice comes from the pot perfectly done, of a clear white, with each grain *firm* and *distinct*, and swelled to double its original size. Salt, of course, is to be added. Rice prepared in this way should *not be stirred* much in boiling, or will it become gluey; a large open fork passed through it once or twice be sufficient.

2. Rice pie.—Chief ingredients: a pint of rice and a fat, tender fowl First boil the fowl in water enough to cook the rice, according to the rule just given. When the fowl is done take out the larger bones and cut it into small pieces. Spread a layer of the cooked rice on the bottom of a deep pan, and on it place a layer of the fowl, with butter and eggs mixed, and with black pepper and spices to suit. Alternate these layers until the pan is full, having a layer of rice at top, on which pour a mixture of butter and egg, and set the whole to "browning" in an oven or on the fire; then serve it at table in the vessel in which it is last cooked. This dish somewhat resembles in richness the celebrated pilua of the Turks.—*Good Health.*

---

* It is at this stage, the water being poured off, that it is sometimes prepared for invalids by pouring in milk and boiling with sugar, spices, or other flavors, to suit the taste.

